# Reoperation for bleeding in an elective cardiac surgical population - Does it affect survival?

**DOI:** 10.34172/jcvtr.2021.34

**Published:** 2021-05-29

**Authors:** Saddiq Mohammad Qazi, Kristian Kandler, Peter Skov Olsen

**Affiliations:** Department of Cardiothoracic Surgery, Rigshospitalet, Copenhagen, Denmark

**Keywords:** Cardiac Surgery, Postoperative Bleeding, Transfusions

## Abstract

***Introduction:*** Earlier studies have shown that re-operation for bleeding after cardiac surgery is associated with increased mortality and morbidity in both acute and elective patients. The aim of the study was to assess the effect of re-operation for bleeding on short- and long-term survival and the causes of re-operation on an exclusively elective population.

***Methods:*** This was a single-center, retrospective study conducted at the Department of Cardiothoracic Surgery at Copenhagen University Hospital. Rigshospitalet, Denmark. We included all elective patients undergoing first-time coronary bypass, valve surgery or combinations hereof between January 1998 and February 2014. Data was obtained from the electronic patient records on demographics, cardiological risk profile, blood transfusion and surgical record.

***Results:*** A total of 11813 patients were included in the analysis of whom 626 (5.3%) patients underwent re-operation for bleeding. Patients were divided into two groups; non re-operated (NRO) and re-operated(RO). Baseline characteristics were comparable. Median survival was lover in the RO group (142 vs 160months (*P* = 0.001)). Morbidity and 30 day mortality was significantly higher in the RO group. Cox-regression analysis showed a significantly increased age-adjusted risk of death in the RO group (HR 1.21(1.07-1.37). *P* = 0.003). In 85% of the patients the site of bleeding was found during the re-operation.

***Conclusion:*** We found both short and long-term survival to be lower in the RO group. A surgical cause for re-operation was found in the majority of cases. The study shows the importance of meticulous hemostasis during cardiac surgery.

## Introduction

Early postoperative bleeding is a serious complication after cardiac surgery, as it can lead to haemodynamic instability, cardiac tamponade, blood transfusions and ultimately a need for re-sternotomy and a worse outcome for the patient. In previous studies on reoperation for bleeding many factors have been shown to affect mortality and morbidity. The reoperation in it self has been shown to increase mortality more than fourfold.^1^ A further detrimental effect of transfusions of red blood cells (RBCs) has in retrospective studies been associated with an up to four times increase in mortality, both on short- and long-term follow-up,^2,3^ though this detrimental effect has been elusive in prospective randomized trials. However, the bulk of existing evidence comes from studies with both elective and urgent patients, and where the latter cases could be associated with ongoing anti-platelet and coagulation therapy, more severe diagnosis (e.g. aortic dissection) and a higher perioperative mortality.^4,5^ The aim of this study was to assess the effect of reoperation for bleeding on an elective population, as this has not been published before. We hypothesized that re-operation for bleeding increased short- and long-term mortality.

## Materials and methods

### Design

This was a single-center retrospective study conducted at the Department of Cardiothoracic Surgery at Rigshospitalet, Copenhagen, Denmark. We included all elective patients undergoing first-time cardiac surgery between January 1998 and February 2014. We included CABG, valve (mitral, aortic and tricuspid) procedures and combinations hereof. Exclusion criteria were age under 18 years, congenital heart disease, non-elective surgery, previous cardiac surgery, more than three procedures, coronary re-implantation (f.ex. Bentall procedures), aortic arch surgery and foreign citizens (follow-up not possible).

The primary outcome was long-term survival. Secondary outcomes included morbidity and surgical findings during reoperation.

The preoperative and surgical data was obtained from the Patient Analysis and Tracking System (PATS) database (Axis Clinical Software Inc., Portland, USA) containing surgical and cardiological data. Follow-up was conducted via PATS, which automatically collects date of death from the National Patient Registry.

Data on the use of blood products was obtained from the local blood bank at our institution. We recorded all red blood cell transfusions (RBCs), thrombocyte pools (from 4 donors) and plasma units within 30 days postoperative. Our transfusion cutoff was 7.25 g/dL during the study period, however in the acute setting of a reoperation for bleeding, the hemoglobin concentration is rarely considered. In these situations, hemodynamics assed by invasive blood pressure monitoring, lactate level development, central venous oxygen saturation and pressure along with cardiac ultrasound performed by a cardiologist

The resulting composite database was internally validated for duplicates and non-matched patients.

### Criteria for reoperation

The decision to reoperate was made by the surgeons on call. At our center we adhere to the Kirklin/Barrat-Boyes criteria for reoperation or on the type of bleeding such as sudden massive bleeding, or haemodynamic instability; reoperation is initiated if drainage output is above 500 mL during the first hour, more than 400 mL during each of the first 2 hours, more than 300 mL during each of the first 3 hours, or more than 1000 mL in total in the first 4 hours.^6^

### Statistics analysis

Continuous data are presented as means ± standard deviations (SD) when normally distributed or median (range) when the distribution is skewed. Continuous variables were compared by Student’s *t*-test, when normally distributed. Skewed continuous data were analysed using the Mann-Whitney *U* test. Categorical variables were compared by Pearson´s chi-squared test, and Bonferroni correction for multiple testing. Based on the significant parameters in the univariate analysis, was included in a binary logstic regression model. Excepted from this was double procedures, as to eliminate risk of multicollinearity. In the binary logistic regression analysis, a backwards stepwise model selection was used with the criteria of *P* ≥ 0.10 for removal of the covariate from the model. The included variables included had a normal distribution.

Survival analysis was performed using Cox proportional hazard analysis adjusted for age and the log rank test.

Differences was considered significant when *P* < 0.05. All calculations were done using SPSS 21.0 (IBM, New York, USA).

## Results

A total of 12439 patients were included in the analysis, of whom 626 (5.6%) patients underwent reoperation for bleeding. Patients were divided into two groups; non-reoperated (non-RO) and reoperated (RO). The baseline characteristics of the two groups are shown in Table 1. Table 2 shows the character of the procedures.

**Table 1:** Baseline characteristics

	**Non-reoperated** **n=11187**	**Reoperated** **n=626**	***P*** ** value**
Patient demographics			
-Age (years)	65.8 (+/- 10.5)	66.9 (+/-10.9)	0.007
-Gender (males)	8423 (75%)	471 (75%)	0.98
-BMI	27.1 (+/- 4.5)	26.2(+/- 4.2)	0.001
Hypercholesterolimia	5402 (48.3)	271 (43,3)	0.015
Hypertension	4581 (40.9)	231 (36.9)	0.045
Diabetes, all types Smoking, active	1880 (16.8) 2221 (19.9)	70 (11.2) 112 (17.9)	0.0002 0.12
Peripheral arterial disease	880 (7.9)	43 (6.9)	0.77
-Se-creatinine. micromol/L Dialysis. no	96 (+/-66.0) 67 (0.6)	102 (+/-69.0) 10 (1.6)	0.08 0.006
Family history of IHD	2399 (21.4)	118 (18.8)	0.11
Prior stroke/TCI	802 (7.2)	47 (7.5)	0.85
MI (< 12 months)	102 (0.9)	6 (1.0)	0.92
NYHA class NYHA I NYHA II NYHA III NYHA IV	1008 (9.0) 2560 (22.9) 1179 (10.5) 72 (0.6)	59 (9.4) 140 (22.4) 65 (10.4) 7 (1.1)	0.82 0.72 0.90 0.25
CCS class CCS I CCS II CCS III CCS IV No angina	1025 (9.2) 2839 (25.4) 1186 (10.6) 210 (1.9) 3302 (29.5)	51 (8.1) 132 (21.1) 80 (12.8) 14 (2.2) 189 (30.2)	0.39 0.01 0.12 0.64 0.85
Preoperative LVEF			
0-29%	216 (1.9)	9 (1.4)	0.45
30-49%	700 (6.3)	38 (6.1)	0.87

Abbreviations: BMI, body mass index; MI, myocardial infarction; NYHA, New York Heart Association score; CCS, Canadian Cardiovascular Society grading; LVEF, left ventricular ejection fraction; IHD, ischaemic heart disease;TCI, transitory cerebral ischaemia Baseline characteristics and preoperative data for the two groups is shown in this table. Continous data are presented as mean +/- standard deviation or median (range). Categorical data are presented as number (percentage of total in subgroup). Level of significance after Bonferroni correction is 0.002

**Table 2:** Operative data

	**Non-reoperated**	**Reoperated**	***P*** ** value**
	**n=11187**	**n=626**	
*Single procedures*			
- CABG	6590	313	
- Valve	2710	173	
*Single procedures total*	9300 (83,1%)	486 (78,7%)	0.0001
*Double procedures*			
- Valve+CABG	1276	99	
- Valve + other	497	37	
- CABG + other	114	4	
*Multiple procedures total*	1887 (16,9%)	140 (22,3%)	0.0005
ECC time, min	97 (+/- 36)	105(+/-39)	0.001

Abbreviations: CABG, coronary artery bypass graft; ECC, extracorporal circulation Type of elective surgery and the distribution of single and multiple procedures for the two groups is shown. Continous data are presented as mean +/- standard deviation or median (range). Categorical data are presented as number (percentage of total in subgroup)

Our logistic regression model found that diabetes (OR 1.35 (CI 1.02-1.78)), BMI (OR 0,96 (CI 0.94-0.99)) and ECC duration (OR 1.005 (CI 1.003-1.007)) were independently associated with reoperation for bleeding. Age was not found to be independently associated with reoperation for bleeding.

**Figure 1:**
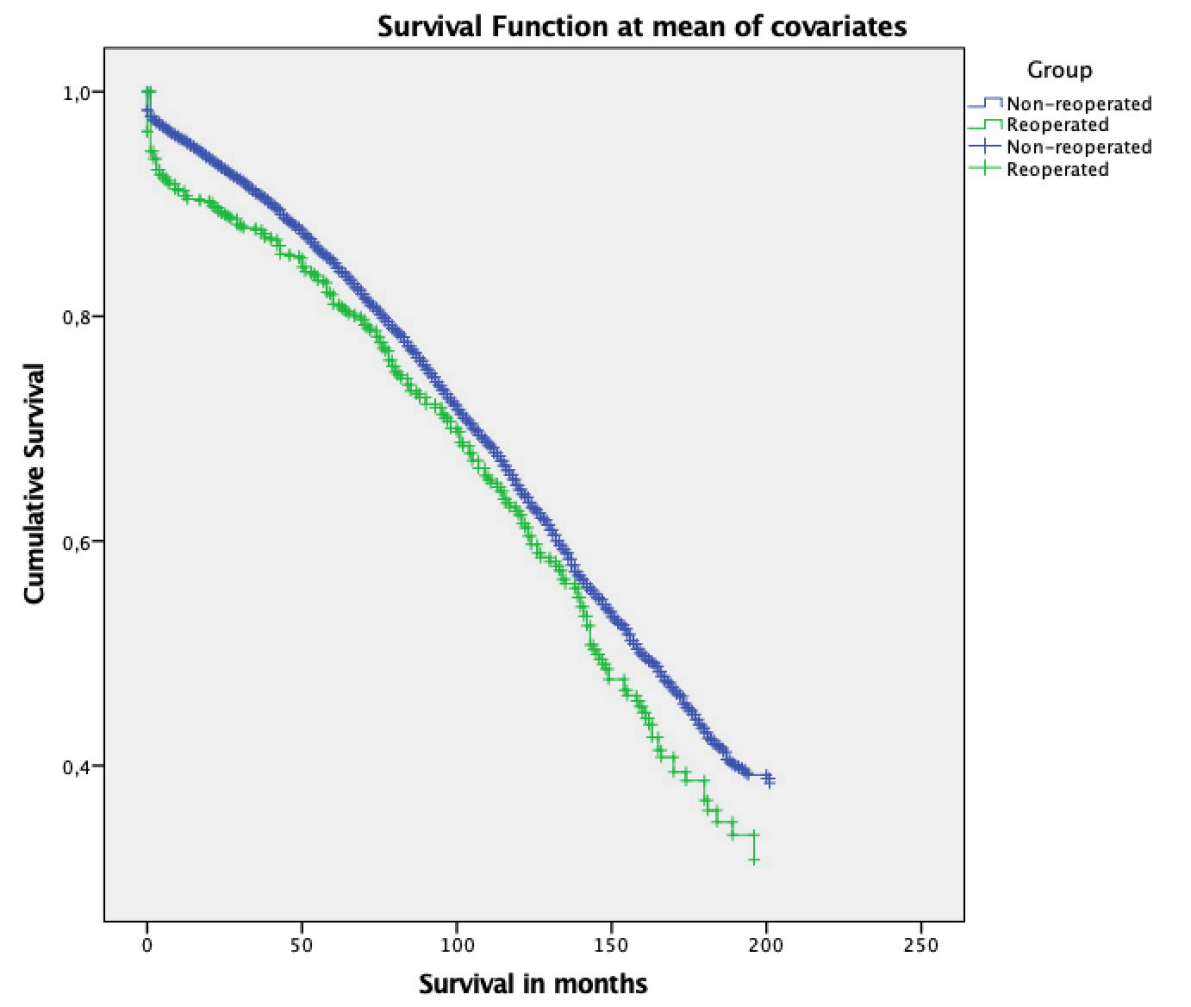
Age adjusted Cox-regression survival analysis is shown for the study period. Individual cross-marks shows death of a patient in the respective groups.

We found a significantly higher 30-day mortality and overall mortality in the RO group (Figure 1). There were significant differences between the two groups on almost all postoperative parameters (Table 3). Furthermore, significantly more transfusions were utilized in the RO group.

**Table 3:** Postoperative data

	**Non-reoperated** **n=11187**	**Reoperated** **n=626**	***P *** **value**
Follow-up			
Median survival, months (95% CI)	160 (154-165)	142 (125-138)	0.001
Overall mortality	3346 (29.9)	261 (41.7)	0.001
Postoperative complications			
30 day mortality	253 (2.2)	30 (4.8)	0.001
1-year mortality	545 (4.9)	65 (10.4)	0.001
5-year mortality	3214 (28.7)	243 (38.9)	0.001
Myocardial infarction	168 (1.5)	12 (1.9)	0.45
Atrial fibrillation Stroke Deep wound infection	4441 (39.7) 134 (1.2) 75 (0.7)	304 (48.6) 17 (2.7) 15 (2.4)	0.0003 0.0005 0.0001
Renal failure – dialysis	233 (2.1)	49 (7.8)	0.0001
Multi organ failure	98 (0.9)	22 (3.5)	0.0001
ICU stay, days	1.3 (+/- 2.4)	2.4 (+/- 4.1)	0.001
Ventilator > 48h	393 (3.4)	91(14.0)	0.001
Transfusions			
RBCs	1.3 (+/-3.0)	8.0 (+/-8.8)	0.001
Thrombocytes	0.2 (+/-0.8)	2.2 (+/-2.8)	0.001
Plasma	0.4 (+/- 2.4)	5.0 (+/-7.1)	0.001

Abbreviations: CI, confidence interval; RBCs, red blood cells Mortality, major complications and number of transfusions is presented for the two groups. Continous data are presented as mean +/- standard deviation or median (range). Categorical data are presented as number (percentage of total in subgroup)

Cox-regression analysis showed a significantly increased age-adjusted risk of death in the RO group (HR 1.21 (1.07-1.37). *P* = 0.003).

In 258 (41% of all RO patients, ) patients the surgical findings were given in the database. The bleeding site database was an addition to our PATS database that came late in the study population. In 40 patients (16%) the reason given as ”no surgical cause found” or ”coagulopathy”, in the remaining 218 patients (85%) a surgical bleeding site was found (Table 4).

**Table 4:** Source of bleeding

**Source of bleeding**	**N** **(n=258)**
Anastomosis	34 (13%)
Mammary artery or graftsite	19 (7%)
Cannulation	25 (10%)
Sternum	44 (17%)
Multiple sites	77 (30%)
Other	19 (7%)
Not identified	33 (13%)
”Medical bleeding”	7 (3%)

The site of bleeding is shown, as registered by the surgeon after the reoperation for bleeding.

## Discussion

Undergoing a reoperation for bleeding as well as receiving blood transfusions after cardiac surgery has been shown to be associated with a higher morbidity and mortality in a general cardiac surgery population, which also includes acute and sub-acute cases.^1,7^ This study examined if a reoperation and the associated complications affected survival in a group of elective patients undergoing cardiac surgery. We found a similar pattern with a significantly increased mortality as well as morbidity in patients undergoing reoperation, and a higher requirement of RBC transfusions.

In our study the RO group received five times as many units of RBCs compared to the non-RO group, and there are previous studies to support that this has a detrimental effect on the survival.

Our transfusion cutoff was 7.25 g/dL during the study period, however in the acute setting of a reoperation for bleeding, the hemoglobin concentration is rarely considered. In these situations, hemodynamics assed by invasive blood pressure monitoring, lactate level development, central venous oxygen saturation and pressure along with cardiac ultrasound performed by a cardiologist was essential in the decision-making for reoperation and transfusions.

Koch et al examined a population of 11,963 isolated CABG patients and found a significantly increased and dose-dependent mortality when RBC transfusion was given (OR = 1.77 for a single RBC-transfusion),^8^ and these findings are supported by multiple studies.^9-13^ However all these studies included non-elective patient groups, as opposed to this study.

There is also evidence to support that transfusions can have a detrimental effect on long term survival.^14^ However our data foremost shows a rapid decline in survival in the immediate postoperative period in the RO group (Figure 1), a distribution similar to that found in other studies.^11^ The increased early mortality is most likely caused by an increase in early postoperative complications, as can be seen in Table 3. This is reflected in the survival curve, as the RO group has an initial drop, in the long term our curves are similar and seems parallel. This could imply an immediate effect of reoperation and complications, without any detrimental effects on the long term.

Although the relationship between transfusions and mortality is well-documented in retrospective studies, the link has been somewhat elusive in randomised controlled studies. Patel et al in their meta-analysis did not find a link between a liberal transfusion-strategy and increased mortality in a population of patients undergoing cardiac surgery.^15^ The discrepancies between prospective and retrospective studies could reflect that the relationship between transfusions and survival is heavily confounded, and difficult to adjust for, as multiple transfusions are expected to be given to anemic, bleeding and co-morbid patients, all of which could affect survival.^13^ The later “The Transfusion Requirements in Cardiac Surgery (TRICS)” randomized trials did not find significant differences between a liberal or restrictive transfusions in mortality outcomes, even at 6 months.^16^ This makes interpretations of the significant and considerabel effects of transfusions on mortality in retrospective studies even more obscure, and could imply shortcomings of statistical models to exclude the effect of confounding.

The reoperation for bleeding has, in itself, been associated with an increase in mortality. Ranucci et al reported twice the mortality compared to the mortality predicted by EUROscore.^7^ Moulton et al reported an odds ratio of 4.4 on mortality in a low-risk elective CABG-population, when reoperation was needed.^17^ In addition to a significantly higher mortality, our data shows an increased incidence of acute kidney injury, infections, stroke and atrial fibrillation in the RO group. This could reflect the additional surgical stress, inflammation, possible anemia, hypoxia, prolonged ventilation and exposure to microbes associated with reoperation. The long-term mortality in this study was comparable to a previous study with up to 30 years follow-up.^18^

Both our populations had similar pre-operative risk profiles, however there was a large difference in the postoperative outcomes between the two groups. Furthermore, the findings during reoperation were predominantly surgical bleeding (83.4%) and to lesser extent coagulopathy (16.4%), which is similar to earlier reports.^19^ This points towards a predominantly insufficient surgical haemostasis at the primary operation. However, the initial source of bleeding which causes the need for reoperation can be difficult to assess due to factors such as removal of steelwires, bleeding because of re-opening and new onset of coagulopathy. Our study is conducted at one center, on a very large number of patients, with complete and long-term follow-up.

Our study is primarily limited by its retrospective design. Our inclusion period spans more than a decade; however, we do not believe this has affected our outcome. A propensity match study was considered but advised against by our statistician.

We did not have information on preoperative anticoagulants and other drugs. However, during the study period it was the policy of our center to stop treatment with acetylsalicylic acid and clopidogrel for five days prior to surgery, and warfarin three days prior to elective surgery.

## Conclusion

In this study we found a mortality hazard ratio of 1.21 in patients undergoing reoperation for bleeding after elective cardiac surgery. The RO group had a significantly higher number of complications, translating into lower short and long-term survival. As a surgical cause for reoperation was found in the majority of cases, stressing the need for careful haemostasis during the operation to avoid a possible initiating event for other complications.

## Competing interest

The authors have no competing interests.

## Ethical approval

Due to the retrospective nature of this study, an ethical and legal approval for data collection was approved by the Danish Patient Safety Autority, approval number: 2012-58-0004.

## Funding

Due to the retrospective nature of this study, no funding was needed.
